# Enhancing oral squamous cell carcinoma detection: a novel approach using improved EfficientNet architecture

**DOI:** 10.1186/s12903-024-04307-5

**Published:** 2024-05-23

**Authors:** Aradhana Soni, Prabira Kumar Sethy, Amit Kumar Dewangan, Aziz Nanthaamornphong, Santi Kumari Behera, Baishnu Devi

**Affiliations:** 1 Department of Information Technology, Guru Ghasidas Vishwavidyalaya, Bilaspur, India; 2 Department of ECE, Guru Ghasidas Vishwavidyalaya, Bilaspur, C.G India; 3 College of Computing, Prince of Songkla University, Phuket campus, Phuket, Thailand; 4 Department of Computer Science and Engineering, VSSUT, Burla, India

**Keywords:** Oral carcinoma, OSCC, Histopathological images, Classification, EfficientNet

## Abstract

**Problem:**

Oral squamous cell carcinoma (OSCC) is the eighth most prevalent cancer globally, leading to the loss of structural integrity within the oral cavity layers and membranes. Despite its high prevalence, early diagnosis is crucial for effective treatment.

**Aim:**

This study aimed to utilize recent advancements in deep learning for medical image classification to automate the early diagnosis of oral histopathology images, thereby facilitating prompt and accurate detection of oral cancer.

**Methods:**

A deep learning convolutional neural network (CNN) model categorizes benign and malignant oral biopsy histopathological images. By leveraging 17 pretrained DL-CNN models, a two-step statistical analysis identified the pretrained EfficientNetB0 model as the most superior. Further enhancement of EfficientNetB0 was achieved by incorporating a dual attention network (DAN) into the model architecture.

**Results:**

The improved EfficientNetB0 model demonstrated impressive performance metrics, including an accuracy of 91.1%, sensitivity of 92.2%, specificity of 91.0%, precision of 91.3%, false-positive rate (FPR) of 1.12%, F1 score of 92.3%, Matthews correlation coefficient (MCC) of 90.1%, kappa of 88.8%, and computational time of 66.41%. Notably, this model surpasses the performance of state-of-the-art approaches in the field.

**Conclusion:**

Integrating deep learning techniques, specifically the enhanced EfficientNetB0 model with DAN, shows promising results for the automated early diagnosis of oral cancer through oral histopathology image analysis. This advancement has significant potential for improving the efficacy of oral cancer treatment strategies.

## Introduction

Oral cancer is the eighth most common type of cancer in the world. Each year, approximately 274,000 new cases are diagnosed. Most individuals with oral cancer live in developing countries. Cancer has become one of the main causes of death in India. Oral cancer has a higher mortality rate than other types of cancer. It is the most common cancer in men and the third most common cancer in women. It accounts for 17% of all cancers in men and 10.5% of all cancers in women. Studies have shown that less than 65% of primary care centers in low- and middle-income countries can receive good pathology services [1–3]. Oral cancer can affect the lips, mouth, and back of the throat. When this happens, the structural layers and membranes in the mouth and throat are lost. Oral malignancies include OSCC, salivary gland, verrucous, and lymphoepithelial carcinoma. Most carcinomas are caused by OSCC [4, 5]. The total mortality rate of OSCC patients has not greatly decreased despite the use of various treatment modalities, which is solely because early identification and diagnostic efforts have not been made. Doctors should examine any worrisome lesions that may be malignant and then recommend a biopsy. Under a microscope, slides containing biopsy sections are checked for abnormalities that deviate from typical cell configurations in size and shape. Malignant squamous cells differ significantly from one another in terms of morphology at histopathological stages and are larger than normal cells. It is extremely important and accurate for a highly skilled and experienced physician to make a confirmatory diagnosis of oral cancer from these data. Nevertheless, the entire manual process of manually interpreting each portion of a slide and analyzing malignant cells takes too much time and is subject to human mistakes [6, 7]. Owing to the abovementioned factors, computer-aided diagnostic (CAD) procedures may help doctors analyze features more quickly and accurately while saving time. The goal is to identify cancer at an early stage so that it may be treated promptly, reducing the risk of morbidity and mortality. In addition, in most cases of cancer, CAD systems can detect it, which implies that pathologists have attempted to detect more cases. In contrast to late detection, which results in a 30% survival rate, early detection of oral cancer increases survival rates to 80% [8, 9].

Recent advances in artificial intelligence have begun to influence the medical field. CNNs have become prominent among these DL approaches because of their excellent accuracy for image classification, particularly for texture classification tasks. Several strategies for diagnosing cancer and COVID-19 have been proposed and developed based on DL. It has been demonstrated that DL techniques offer higher accuracy. Additionally, the transfer learning method is commonly used to classify medical images, improving the outcomes of DL approaches. The usefulness of DL methods, such as histological or real-time oral cavity imaging, in classifying oral lesions from medical images has also been demonstrated by recent research. Several studies have been conducted to diagnose oral cancer based on machine learning and DL using histopathological images. A lightweight DL-CNN, EfficientNet-B0, was created by Fahed Jubair et al. to conduct a binary classification of 716 real-time clinical images into potentially cancerous or benign images. The proposed DL-CNN model achieved an accuracy of 85.0% [10]. “Nandita et al. proposed an ensemble DL-CNN model combining two models, i.e., ResNet-50 and VGG-16. The accuracy of this ensemble model, which was trained using a dataset of enhanced oral lesion images, was 96.20% [11]”. “For the multiclass grading method of OSCC, Das et al. proposed a DL classification model to classify OSCC into four classes. First, pretrained models, such as AlexNet, VGG-16, VGG-19, and ResNet-50, are trained through the transfer learning approach. They achieved the highest classification accuracy of 92.15% with ResNet-50 [12]”. Fu et al. used 44,409 total biopsy-proven OSCC photographic images and conventional clinical features to classify OSCC using cascaded DL. The sensitivity of the DL methods used was 94.90% [13]. They implemented a two-stage model to identify oral lesions and classify them into three categories—benign, OMD, and carcinoma. Tanriver et al. presented a DL EfficientNet-B7 model for detecting oral malignant disorders or OMDs. The tumor pathology department at Istanbul University’s oncology institute provided the oral, photographic dataset with lesions. The model’s highest level of accuracy recorded was 92.9% [14]. Mohammed Zubair et al. suggested a DL model utilizing the transfer learning approach to categorize five forms of oral precancerous lesions from annotated images and recognize the first stage of oral cancer. The classification accuracy was 97.00% for ResNet50 and 98.00% for VGG-19 [15]. “Gupta et al. proposed a deep learning CNN model to classify images of dysplastic cells from the oral squamous epithelium layer. The suggested framework divides dysplastic cell images into four categories: normal, mild, moderate, and severe dysplastic cells. The dataset included 2557 photos obtained from 52 patients. The suggested model’s findings reveal a training accuracy of 94.6% and a testing accuracy of 90.22% [16]”. Rachit Kumar Gupta et al. proposed a DL-based CNN framework for classifying dysplastic tissue images. The CNN model categorizes the presented images into four groups: normal, mild, moderate, and severe dysplastic tissue. Biopsy samples from 52 patients, totaling 2688 images, were taken. The training accuracy was 91.65%, while the testing accuracy was 89.3% [17]. Song et al. created a portable smartphone-based oral inspection tool and showed how DL approaches can effectively identify dual-modal photos to identify oral cancer. The fusion of white light and fluorescence images is used in an image classification technique that feeds data to a DL-CNN. For the VGG-CNN-M network, the authors obtained a validation accuracy of 86.90% [18]. Sharma et al. studied the clinical pictures of patients with OSCC and OPMDs. These images were analyzed in comparison to images of the normal oral mucosa. Transfer learning employing different pretrained CNN architectures was used for picture categorization. The accuracy for VGG19 was 76%, that for VGG16 was 72%, that for MobileNet was 72%, that for InceptionV3 was 68%, and that for ResNet50 was 36%. VGG19 performed better in the current investigation than did the other models [19].

Previous studies have attempted to utilize deep learning models to classify oral lesions, including oral squamous cell carcinoma (OSCC); however, notable drawbacks need to be addressed. These limitations include reliance on small or limited datasets, the use of single-modal data without considering multimodal fusion, and the high computational complexity of some models. Additionally, the lack of comprehensive clinical validation and interpretability in model decision making poses challenges for real-world deployment. However, these studies have also introduced innovations, including comprehensive performance evaluations of multiple CNN models, architectural enhancements, such as dual-attention networks, and rigorous statistical analyses for robust comparisons. Comparative analyses with state-of-the-art approaches have further demonstrated advancements in model performance. Addressing these limitations while building upon innovative methodologies is crucial for enhancing the accuracy, generalizability, and clinical applicability of deep learning-based oral lesion classification systems.

In this study, we were mainly interested in classifying OSCC based on cellular-level changes due to carcinoma, which supports clinical decisions. Therefore, we developed an automated OSCC classification method using histopathological images in this study. As a result, we demonstrated that a computerized classification method could be used for oral carcinoma classification, i.e., benign or malignant.

This study makes significant contributions to the following areas.

- We conducted a comprehensive performance evaluation of 17 CNN models for detecting oral squamous cell carcinoma (OSCC) using histopathological images.
- To identify the most effective CNN model, a two-step statistical analysis involving Duncan’s multiple range test and Wilcoxon signed-rank test was employed.
- The performance of the EfficientNetB0 model was enhanced by introducing a dual-attention network.
- A 5% improvement in accuracy compared with the baseline network was achieved through modifications made to EfficientNetB0.
- A comparative analysis with state-of-the-art approaches was conducted, which demonstrated the superior performance of the proposed model.

The rest of the article is structured as follows: Sect. 2 presents the background study, that is, about deep CNN models and details of the statistical analysis. Section 3 describes the material and methodology. Section 4 describes the findings and discusses the remarkable outcomes. Finally, Sect. 5 concludes the article.

## Background study

This section addresses deep convolutional neural networks and statistical analyses.

### Deep CNN models

**Fig. 1:**
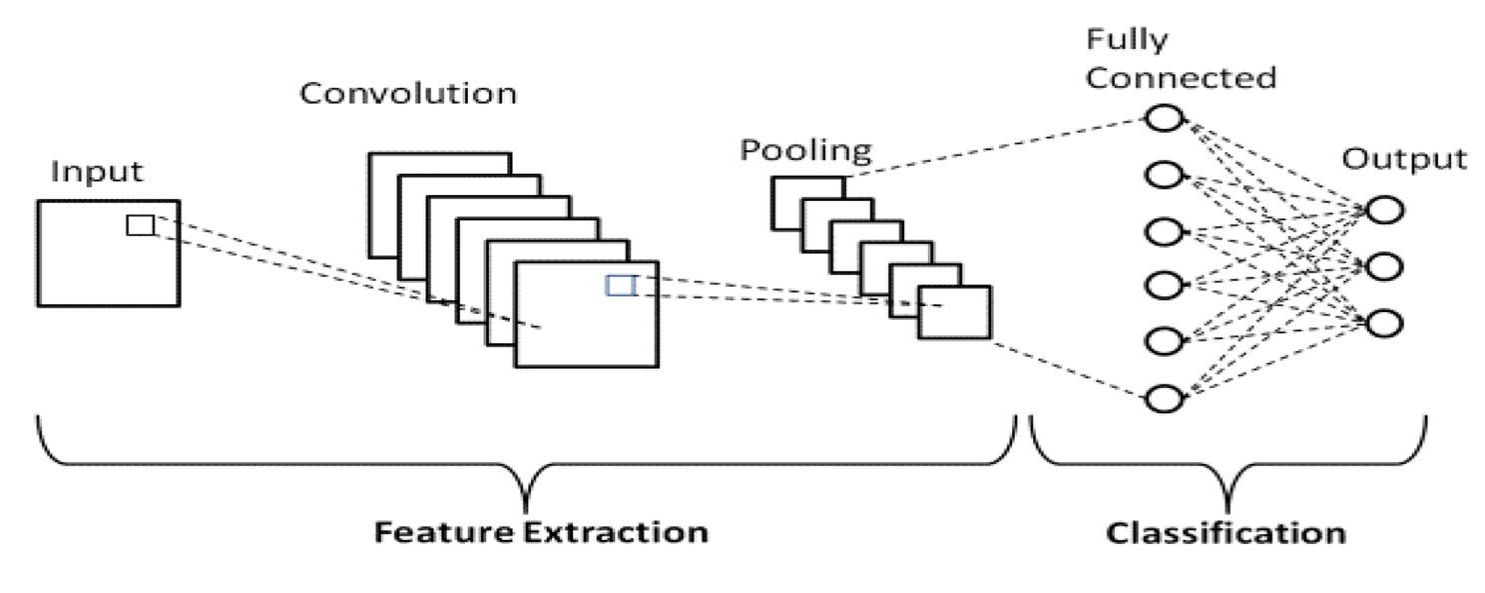
Generalized deep learning CNN model

DL-CNN models have significantly improved the methods currently used for solving various image-based problems, including object recognition, detection, and classification. “A CNN is a DL network constructed using a spatial design that connects a particular region in one layer to a certain region in the next layer. Neurons build the layers, and each layer’s spatial architecture creates a volume of these neurons with a width, height, and depth. Depth and height define the number of neurons, whereas breadth and height dictate their size. The number of stacked layers that make up the entire network can be used to determine the depth of the network. The architecture of a CNN varies based on the usage the architect selects from an infinite number of layer combinations and builds each layer in infinite ways. The three most important layers are completely linked: the convolution, pooling, and fully connected layers [20]”. The DL-CNN model is completed by additional layers, including ReLU, batch normalization, and dropout layers, as illustrated in Fig. 1.

“These layers make learning features from the input photos easier. The convolution layers, composed of several wide, height, and depth filters, extract various characteristics from the input image when fed to a conventional CNN. The width and height determine the filter kernel size, and the depth determines the number of kernels. Each kernel is constructed using parameters that can be learned, which are convolved across the input image and then performed as a dot product to extract features. Size, stride, and padding are a few additional parameters for the convolutional layer. The stride determines how many steps the kernel takes before conducting a convolution operation. The padding regulates the output size from the boundary and layer pixels. The collected characteristics are also given to the pooling layers as input for more effective processing. It is necessary to lower the size of the feature map that the convolution layer created. Although the pooling layers lower the feature map, they perform operations comparable to those of the convolution layers. The two types of layers that are used most frequently are average and maximum pools. As a result, the CNN becomes less computationally difficult as the feature map size decreases. Eventually, the covariant shifts in the intermediate layers are normalized by the batch normalization layer and rectified linear unit (ReLU), enabling improved network convergence. Dropout layers were used to prevent model overfitting. The fully connected layer receives the reduced feature map and applies the SoftMax algorithm to categorize the appropriate classes [20]”.

There are various pretrained DL-CNN models available for image classification. These models include AlexNet, DarkNet19, DarkNet53, GoogleNet, InceptionResNetv2, Inceptionv3, MobileNetv2, NASNetLarge, ResNet18, ResNet50, ResNet101, and EfficientNet. In addition, the DL-CNN models can classify generalized photos that are not part of the ImageNet dataset. We considered all pretrained models, from which we chose EfficientNet and modified it with extra layers for efficient OSCC identification.

- **AlexNet: “**It comprises five convolutional layers, three max-pooling layers, two normalization layers, two fully connected layers, and 1 softmax layer. Each convolutional layer comprises convolutional filters and a rectified linear unit (ReLU) nonlinear activation function. Max pooling is accomplished using the pooling layers. Owing to the existence of completely linked layers, an input size of 224 × 224 × 3 was fixed. If the input image is grayscale, it is converted to RGB by duplicating the single channel to create a three-channel RGB image. AlexNet’s total parameter count was 60 million with a batch size of 128 [21]”.
- **DarkNet 19**: This is a convolutional neural network with a total of 19 layers. A version of the network that has already been trained on more than a million images is stored in the ImageNet database. The network has already been trained to sort photos into 1000 different groups of objects, such as animals, a keyboard, a mouse, and a pencil. Thus, the network learns to represent a wide range of images using many different features. The network also works with images that have a resolution of 256 by 256.
- **DenseNet** is a densely connected convolutional network. Instead of residual connections, the authors proposed dense blocks inspired by ResNet. Like the VGG, the dense block includes successive convolution layers that are connected. Each convolution layer receives all previous layer information. DenseNet had 8,062,504 parameters and a 93.34% top 5 ILSVCR accuracy rating. This network reduces information loss by connecting all layers (especially the deep layers) [22].
- **GoogLeNet**: “It is a 22-layer convolutional neural network. A network that has already been trained can be imported using the Places365 or ImageNet datasets. The network trained on ImageNet divides images into 1000 object categories, including several animals, a keyboard, a mouse, and a pencil. Similar to networks trained on ImageNet, Places365 networks classify photos into 365 distinct place types, such as fields, parks, runways, and lobbies. For a variety of images, these networks have learned several feature representations. The input image size for both untrained networks is 224 by 224 [23]”.
- **InceptionResNetv2: “**It is trained using the ImageNet database. For a variety of photos, the network has learned rich feature representations. The network contains 164 layers, a 299 × 299 input, and generates a list of estimated class probabilities as its output. It is constructed using both the residual connection and the inception structure. Several convolutional filters of various sizes are mixed with residual connections in the Inception-ResNet block. In addition, avoiding the degradation issue caused by deep structures, including residual connections, shortens the training time [24].”
- **Inceptionv3: “**It is a model for image recognition that has been demonstrated to achieve over 78.1% accuracy on the ImageNet dataset. Model components include convolutions, average pooling, maximum pooling, concatenation, dropouts, and fully connected layers. The model uses batch normalization and applies it to activation inputs. SoftMax is used to compute the loss. Inception-v3 is a convolutional neural network design from the inception family that uses label smoothing, factorized 7 × 7 convolutions, and an auxiliary classifier to transport label information down the network along with the use of batch normalization for layers in the side head [24].”
- **MobileNetv2**: “MobileNetV2 has a 32-filter fully convolution layer and 19 residual bottleneck layers. Bottleneck depth-separable convolution with residuals is the foundation of this approach. The input picture resolution and width multiplier are configurable hyperparameters that can be modified for accuracy or performance trade-offs in the architecture. The core network employs 3.4 million parameters and requires 300 million multiple-adds. The model size is 1.7 M to 6.9 M parameters, and the network computational cost is 7 multiply adds to 585 M MAdds [25]”.
- **NASNet Large**: “It is a machine learning model. The key principles differ from those of GoogleNet and may lead to a significant AI breakthrough. The first 414 NASNet-Large layers form the encoder. NASNet has state-of-the-art accuracies of 82.7% top-1 and 96.2% top-5 on ImageNet. The goal was to find the optimal mix of filter sizes, output channels, strides, layers, etc. During each search action, reinforcement learning rewarded accuracy for the searched architecture on the dataset [26]”.
- **NASNetMobile**: “The two primary functions of Nasnetmobile are normal and reduction cells. To attain a higher mAP, NasNet first applies its operations to a small dataset before transferring its block to a large dataset. For better NasNet performance, a customized drop path called a scheduled drop path for effective regularization is utilized. The normal and reduction cells are utilized in the original Nasnet architecture, where the number of cells is not predetermined, and the size of the feature map is dictated by the normal and reduction cells, respectively. Based on the two initial hidden states, a control architecture in Nasnet based on a recurrent neural network (RNN) predicts the whole structure of the network [26]”.
- **ResNet: “**He et al. launched ResNet in 2015, which won the 2015 ImageNet competition with a top-five accuracy percentage of 94.29%. 25,000,000 parameters. ResNet is a deep network with up to 152 layers and a unique residual link that connects the convolutional layers to the ReLU activation layer. The residual connection preserves prior layer weights during backpropagation. This network consists of three layers: ResNet50, ResNet101, and ResNet152. Residual connections allow this network to be used at several levels. Increasing network depth rather than width reduces superfluous parameters. The addition of residual blocks makes the filter size the same, which is this network’s greatest shortcoming. This network’s training requires enormous datasets, making it computationally expensive [27]”.
- **VGG**: “Oxford Visual Geometry Group researchers introduced VGG16 and VGG19 architectures in 2014. The top five accuracy rates of ImageNet 2014 were 91.90% for VGG16. VGG16 has five convolution blocks, three thick layers, and 138,355,752 parameters. Convolutional layers plus a max pooling layer reduce the block output size and noise. The first two blocks have two convolutional layers, and the last three have three. This network’s kernel stride is 1. After the five blocks, a flattened layer was added to transform the 3D vector of the blocks into a 1D vector for the completely connected layers. The first two fully connected layers have 4096 neurons, while the final layer has 1000 neurons. After the completely linked layers, a softmax layer ensures that the output probability summation is one. VGG19 features 19 convolution layers instead of 16 layers. The number of layers increases from 138,357,544 to 143,667,240. The authors claimed that these layers strengthen the architecture and allow it to learn more complex architectures. Sequential blocks reduce spatial information by inserting convolutional layers after each other [28]”.
- **Xception**: “It is a modification to the Inception architecture that uses depthwise separable convolutions in place of the regular Inception modules. The depthwise separable convolution layer and a few shortcut structures are the key components of Xception. Xception features 22.8 million parameters compared to approximately 23.6 million parameters in Inception. It goes well beyond Inception’s guiding concepts. In Inception, the original input was compressed using 1 × 1 convolutions. From each of those input spaces, various filters were applied to each of the depth spaces. The opposite occurs with Xception [29]”.
- **EfficientNet** is based on the basic neural architecture search network from the AutoML MNAS. The network was optimized for accuracy but penalized for computational complexity. A slow inference time penalizes this. Due to FLOPS, the architecture’s mobile inverted bottleneck convolution is larger than that of MobileNet V2. Scaling up this baseline model yields EfficientNets. EfficientNet scales models using compound coefficients. Compound scaling uses a given set of scaling coefficients to consistently scale width, depth, and resolution. EfficientNet has seven multidimensional models using scaling and AutoML, which outperform most convolutional neural networks in terms of accuracy and efficiency [30].

### Statistical analysis

This study analyzes the performance of 16 CNN models and the proposed model using the statistical analysis tool IBM SPSS Statistics 26. Two methods, i.e., the Duncan test and Wilcoxon signed-rank test, were used.

- **Duncan test**

In statistics, David B. Duncan created the multiple comparison method known as Duncan’s new multiple range test (MRT) in 1955. “Duncan’s MRT is a member of the larger group of multiple comparison techniques that compare sets of means using the studentized range statistic qr. This testing was created as a more powerful variation of the Student-Newman‒Keuls approach. The test produces a set of subgroups of means, whereby each subset’s means have been determined to be not significantly different. Duncan’s MRT is particularly protective against false negative (Type II) mistakes while having a greater risk of making false positive (Type I) errors [31]”.

- **Wilcoxon sign test**

“The Wilcoxon signed-rank test is a nonparametric statistical hypothesis test that is used to compare the locations of two populations using two matched samples or to assess the location of a population based on a sample of data. The one-sample version has the same goal as the one-sample Student’s t test. It is a paired difference test for two matched samples, analogous to the paired Student’s t test (also known as the “t test for matched pairs” or “t test for dependent samples”). When population means are unimportant, such as evaluating whether a population’s median is nonzero or whether a sample from one population outweighs a sample from another, the Wilcoxon test can be a helpful substitute for the t test [32]”.

## Materials and methodology

This section addresses the details of the dataset and proposed methodology.

### Dataset

**Fig. 2:**
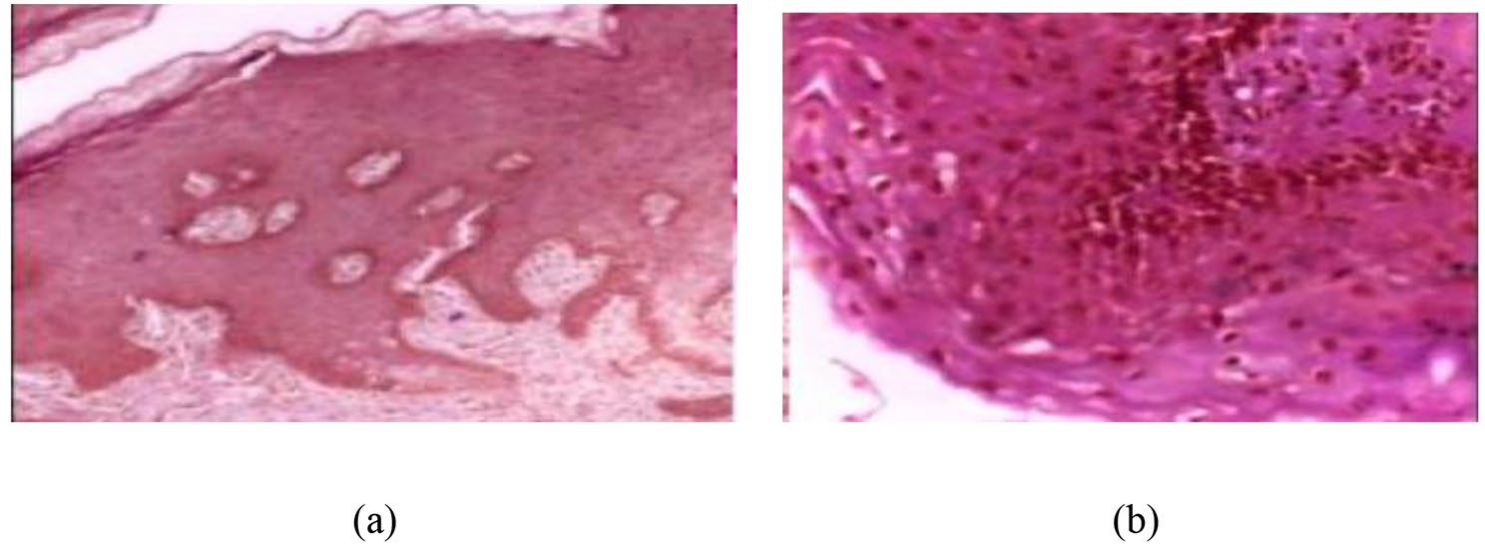
Sample of oral squamous cell histopathological images (**a**) benign (**b**) malignant

There were 1224 total images from 230 patients in this dataset. There are two sets of images, each with a different resolution. “The first collection consisted of 439 OSCC images at 100x magnification and 89 histopathological images of the normal epithelium of the oral cavity. The second group consisted of 495 histopathological images of OSCC tissue at 400x magnification and 201 images of the normal epithelium of the oral cavity. The second group consisted of 495 histopathological images of OSCC tissue at 400x magnification and 201 images of the normal epithelium of the oral cavity. A total of 934 malignant (OSCC) images and 290 normal (benign) oral cavity epithelium images were obtained. Medical professionals collected, processed, and cataloged the slides of tissue stained with H&E. Images were then taken using a Leica ICC50 HD microscope [33]. Histopathological images of oral cancer squamous cell samples are presented in Fig. 2.

### Proposed methodology

This research suggested the detection of OSCC using histopathological images. The methodology comprises three phases. In the first phase, 17 pretrained CNN models were evaluated to detect OSSC. Each CNN model was individually executed 30 times to examine its credibility. Finally, the finding of each execution with seven parametric measures is recorded.

**Fig. 3:**
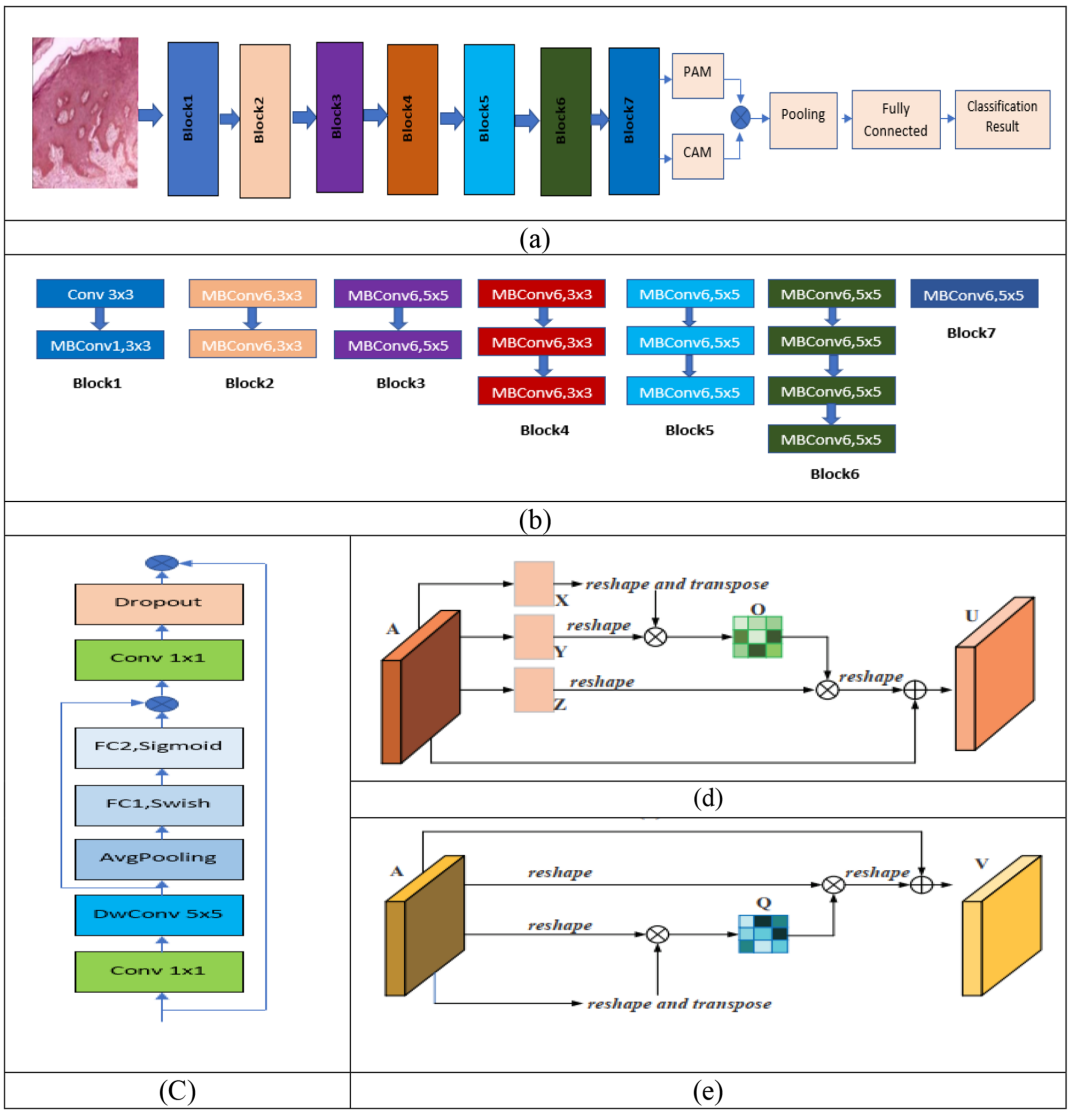
Improved EfficientNet (**a**) basic architecture of improved EfficientNet, (**b**) details of each block of (**a**), (**c**) architecture of MB convolution, (**d**) architecture of PAM, (**e**) architecture of CAM

In the second phase, the statistical analysis was carried out in two steps. In the initial step, the Duncan multiple range test was carried out. From this, the best-performing model is chosen. The Wilcoxon signed-rank test was performed in the second step of the statistical analysis. The high-performance model selected by the Duncan test was used as a reference. Then, the seven parameter measures of the reference model were compared with those of the other 16 CNN models to determine the superior model. In this analysis, the best model obtained was EffcienNetB0, but the accuracy was less than 90%, which is more satisfactory. Hence, we are motivated to improve EffcienNetB0 by modifying its original structure, as illustrated in Fig. 3.

Google published an efficient network in 2019. The baseline network uses a neural architecture search and a scaled model to obtain a series of models. EffcienNetB0 comprises a convolutional layer, an MBconvolution1 layer, an MBconvolution6 layer, a pooling layer, a fully connected layer, and a classification layer.

EfficientNetB0 is a convolutional neural network (CNN) architecture that has gained prominence owing to its efficiency and effectiveness in various computer vision tasks. Below, we outline some of the key strengths of EfficientNetB0 in comparison with other deep learning models.

- Scalability: One of the primary strengths of EfficientNetB0 is its scalable architecture, which is achieved through a compound scaling method. This method optimizes the network depth, width, and resolution simultaneously, resulting in models that are both efficient and accurate across a wide range of computational resources.
- Parameter Efficiency: Compared with other deep learning architectures, EfficientNetB0 achieves superior performance while maintaining a relatively small number of parameters. This efficiency is crucial for applications with limited computational resources, making EfficientNetB0 suitable for deployment on various mobile and edge devices.
- Transfer Learning Capability: Owing to its effectiveness in learning rich feature representations from images, EfficientNetB0 demonstrates strong transfer learning capabilities. Pretrained versions of EfficientNetB0 on large-scale image datasets, such as ImageNet, can be fine-tuned on smaller datasets with specific tasks, leading to improved performance and faster convergence.
- State-of-the-art Performance: EfficientNetB0 consistently achieved state-of-the-art performance across benchmark datasets and computer vision tasks, including image classification, object detection, and segmentation. Its superior performance is attributed to its optimized architecture, which balances model complexity and computational efficiency.
- Generalization Ability: EfficientNetB0 demonstrates robust generalization ability, meaning that it can effectively learn from limited training data and generalize well to unseen data. This is particularly beneficial for medical imaging tasks in which annotated datasets may be limited or expensive to acquire.

In our study, we employed EfficientNetB0 as the backbone architecture for our deep learning model due to these strengths, aiming to leverage its efficiency and performance for classifying oral epithelial lesions.

The modification of the main architecture of EfficientNetB0 is illustrated in Fig. 3(a). The layer of each block is illustrated in Fig. 3 (b). A dual attention network (DAN) is introduced before the fully connected layer. The features extracted from block 7 are fed to pooling through DAN. The blocks are MBConvolution, i.e., MBconvolution1 and MBconvolution6. MBconvolution1 is illustrated in Fig. 3(c); MB convolution refers to an inverted mobile bottleneck [34]. MBconvolution6 is the six-time repeat of MBconvolution1. The input image of the histopathology of OSSC was 300 × 300. The final classification result is processed through a convolution layer, an MB convolution layer, an MB convolution layer, a pooling layer, a fully connected layer, and a classification layer.

The PAM and CAM run in parallel in the DAN. The attention mechanism filters out irrelevant information and prioritizes useful information. The DAN attention mechanism achieves great accuracy by adjusting the relationship between local and global features [35]. Figure 3(d) and 3(e) depict the PAM and CAM, respectively. The position attention module encodes more contextual information into local features, improving their representation capabilities. Following that, we go over the process of adaptively aggregating spatial contexts. As shown in Fig. 3(d), we first feed a local feature A ∈ R^C×H×W^ into a convolution layer to build two new feature maps B and C, where {B, C} ∈ R^C×H×W^. Next, they are reshaped to R^C×N^, where N = H × W is the number of pixels. Next, we perform matrix multiplication on the transpose of C and B and use a softmax layer to compute the spatial attention map S ∈ R^N×N^

1 $${S_{ij}}=\frac{{exp({B_i}.{C_j})}}{{\sum\limits_{{i=1}}^{N} {exp({B_i}.{C_j})} }}$$

where Sji calculates the impact of the i^th^ position on the j^th^ position; the higher the correlation between two places is, the more similar their feature representations are.

Meanwhile, we feed feature A into a convolution layer to create a new feature map D ∈ R^C×H×W^ that we reshape to R^C×N^. The outcome is RCHW when we conduct a matrix multiplication of D and the transpose of S. Last, we multiply it by a scale parameter and execute an elementwise sum operation on the features A to obtain the final result E ∈ R^C×H×W^, as shown

2 $${E_j}=\alpha \sum\limits_{{i=1}}^{N} {\left( {{S_{ji}}.{D_i}} \right)+{A_j}}$$

where it is set to zero at the start and gradually learns to attach a greater weight [36]. Equation 2 shows that the resulting feature E at each place is a weighted sum of the features across all positions and the original features. As a result, it has a global contextual perspective and selectively collects contexts based on the spatial attention map. Similar semantic traits benefit from mutual gains, boosting intraclass compactness and consistency.

Emphasis has now been placed on interdependent feature maps to improve the feature representation of certain semantics. As a result, we create a channel attention module to formally model channel interdependence. The channel attention module topology is depicted in Fig. 3(e). Unlike the position attention module, we calculate the channel attention map X ∈ R^C×C^ straight from the original features A ∈ R^C×H×W^. In particular, we reshape A to R^C×N^ and then execute matrix multiplication on A and its transpose. Finally, a softmax layer is applied to obtain the channel attention map X ∈ R^C×C^.

3 $${x_{ji}}=\frac{{exp({A_i}.{A_j})}}{{\sum\limits_{{i=1}}^{C} {exp({A_i}.{A_j})} }}$$

where x_ji_ is the impact of the i^th^ channel on the j^th^ channel. Furthermore, we conduct matrix multiplication on the transpose of X and A and reshape the output to R^C×H×W^. The result is then multiplied by the scale parameter *β*, and an elementwise sum operation with A is performed to generate the final output E ∈ R^C×H×W^.

4 $${E_j}=\beta \sum\limits_{{i=1}}^{C} {({x_{ji}}.{A_i})+{A_j}}$$

where *β* gradually learns a weight from 0. Equation 4 demonstrates that the final feature of each channel is a weighted sum of all channels’ and original features’ features, which depicts the long-term semantic connections across feature maps. It improves feature discriminability [37].

We applied 17 pretrained DL CNN models—Alexnet, Darknet19, Darknet53, Densenet201, Googlenet, InceptionResNetv2, InceptionV3, Mobilenetv2, NasnetLarge, NasnetMobile, Xception, ResNet18, ResNet50, ResNet101, VGG16, VGG19, and EfficientNet—for OSCC detection. This study used these models to categorize benign and malignant cases from oral lesion histopathology images because they have achieved excellent success in various computer vision and medical image analysis challenges. The best model is then chosen and considered for future comparison.

In summary, the proposed model was executed as follows.

1. Oral squamous cell carcinoma (OSCC) images were collected from clinical databases or medical institutions.
2. Seventeen pretrained deep learning models were used for the classification of benign and malignant lesions in OSCC images.
3. The performance of each model was evaluated using various metrics, including accuracy, sensitivity, specificity, false positive rate (FPR), precision, F1 score, Matthews correlation coefficient (MCC), kappa, and computational time.
4. Statistical analysis, specifically Duncan’s multiple range test, was used to determine the best-performing model among the 17 pretrained models.
5. Further validation of the selected model was performed through additional statistical analysis, such as the Wilcoxon signed-rank test, to confirm its superiority.
6. Both statistical tests confirm that EfficientNetB0 outperforms the other models in terms of classification accuracy and other evaluation metrics.
7. Enhancements to the EfficientNetB0 model, including the incorporation of a dual attention network (DAN) and MobileNet convolutional layers (MBConvolution), were implemented to improve the performance.
8. Sequential execution of the enhanced EfficientNetB0 model on the OSCC image dataset was performed to evaluate its classification performance.
9. The performance of the improved model was assessed using the same set of evaluation metrics to measure any enhancements achieved through the introduction of the dual attention network and MB convolution layers.

## Results and discussion

The proposed methodology was applied to an HP Victus system, which features a 12th generation Intel Core i7 processor and running Windows 11 alongside an NVIDIA GPU, with MATLAB 2022a as the primary programming environment. The enhanced dataset was randomly partitioned into training (80%) and testing (20%) sets to ensure that the classifier could be generalized to unseen patients. By leveraging pretrained convolutional neural network (CNN) models, transfer learning is employed to adapt these models for oral squamous cell carcinoma (OSCC) classification. Hyperparameter settings were carefully selected to optimize the model performance, including an initial learning rate of 0.0001, utilization of the SGDM optimizer, and a mini-batch size of 32. These parameters undergo iterative tuning to achieve optimal classification accuracy and generalization. To classify OSCC as having the best performance, we employed pretrained CNN models in this study. The CNN models used were AlexNet, Darknet19, Darknet53, Densenet201, GoogLeNet, InceptionResNetv2, InceptionV3, Mobilenetv2, NasnetLarge, NasnetMobile, Xception, ResNet18, ResNet50, ResNet101, VGG16, VGG19, and EfficientNetB0. The results presented in this work are the average of 30 independent runs. The performance of CNN classifiers is measured using confusion matrix measures, i.e., the accuracy (Acc), sensitivity (Sen), specificity (Spe), precision (Pre), FPR, F1 score, kappa values, and computational time. Statistical analysis was carried out to choose the best model. The statistical analysis comprised two steps. The Duncan multirange test was applied in the initial step, as shown in Tables 1 and 2. The same subset exhibits similar performance, although they may have distinct characteristics or features (since superscript letters are identical columnwise, i.e., ‘a’). A *p* value between 0 and 1 is frequently used to indicate the degree of statistical significance. The p values for comparing the categorization methods were all greater than the typical value of 0.05 based on the findings of the statistical study. As a result, it cannot be concluded that there is a significant difference between the methodologies, which is the null hypothesis. Tables 1 and 2 show that EfficientNetB0 is significantly different from the others in terms of seven confusion matrix measures.

**Table 1:** Shows accuracy, sensitivity, specificity, and precision of CNN Model in duncan statistical test

Model Name	Accuracy	sensitivity	specificity	precision
AlexNet	0.7227^ab^	0.6891^abc^	0.7564^a^	0.7871^abc^
DarkNet19	0.7983^efg^	0.7758^bcdef^	0.8208^ab^	0.8341^bcde^
Darknet53	0.8053^fgh^	0.8044^cdef^	0.8061^ab^	0.8288^bcde^
Densenet201	0.8233^gh^	0.8210^def^	0.8257^ab^	0.8418^de^
Google Net	0.6971^a^	0.6353^a^	0.75884^a^	0.7828^ab^
Inceptionresnetv2	0.8180^fgh^	0.8032^cdef^	0.8328^ab^	0.8438^de^
Inceptionv3	0.8071^fgh^	0.8023^cdef^	0.8120^ab^	0.8225^abcde^
Mobilenetv2	0.8337^h^	0.8424^ef^	0.8251^ab^	0.8370^cde^
NasNet Large	0.7904^ef^	0.7624^bcdef^	0.8184^ab^	0.8324^bcde^
Nasnet Mobile	0.7695^de^	0.7436^abcde^	0.7955^ab^	0.8089^abcd^
Resnet18	0.7589^cd^	0.7372^abcde^	0.7806^ab^	0.8008^abcd^
Resnet50	0.7178^ab^	0.6808^ab^	0.7548^a^	0.7954^abcd^
Resnet101	0.7540^cd^	0.7487^abcde^	0.7593^a^	0.7941^abcd^
Vgg16	0.7605^cd^	0.7379^abcde^	0.7832^ab^	0.8070^abcd^
Vgg19	0.7314^ab^	0.7205^abcd^	0.7423^a^	0.7753^a^
Xception	0.8158^fgh^	0.8227^def^	0.8089^ab^	0.8247^abcde^
Efficient Net B0	**0.8666** ^**i**^	**0.8772** ^**f**^	**0.8561** ^**ab**^	**0.8696** ^**e**^

**Table 2:** Shows FPR, F1 score, MCC, kappa, comp time of CNN model in duncan statistical Test

Model Name	FPR	F1 Score	MCC	Kappa	Comp time
AlexNet	0.2435^b^	0.6701^ab^	0.4893^ab^	0.4455^ab^	20.18^c^
DarkNet19	0.1791^ab^	0.7882^def^	0.6182^fg^	0.5967^fgh^	14.64^a^
Darknet53	0.1938^ab^	0.7939^def^	0.6347^fgh^	0.6106^ghi^	75.46^g^
Densenet201	0.1742^ab^	0.8167^efg^	0.6661^gh^	0.6360^hi^	116.81^k^
Google Net	0.2411^b^	0.6444^a^	0.4452^a^	0.3942^a^	20.84^c^
Inceptionresnetv2	0.1671^ab^	0.8068^efg^	0.6542^gh^	0.6360^ghi^	91.21^i^
Inceptionv3	0.1879^ab^	0.8007^defg^	0.6283^fgh^	0.6143^ghi^	44.12^e^
Mobilenetv2	0.1748^ab^	0.8339^fg^	0.6768^h^	0.6467^h^	23.70^d^
Nasnet Large	0.1815^ab^	0.7786^cdef^	0.6050^ef^	0.5809^fg^	334.23^m^
Nasnet Mobile	0.2044^ab^	0.7504^cde^	0.5642^de^	0.5391^ef^	155.35^l^
Resnet18	0.2193^ab^	0.7384^cd^	0.5484^d^	0.5179^de^	18.27^b^
Resnet50	0.2451^b^	0.6667^ab^	0.4853^ab^	0.5179^cd^	20.21^c^
Resnet101	0.2406^b^	0.7352^cd^	0.5422^cd^	0.5081^de^	91.06^i^
Vgg16	0.2167^ab^	0.7349^cd^	0.5549^d^	0.5211^de^	90.62^i^
Vgg19	0.2576^b^	0.7130^bc^	0.5005^bc^	0.4629^cd^	112.20^j^
Xception	0.1910^ab^	0.8111^efg^	0.6475^fgh^	0.6317^ghi^	84.58^h^
Efficient Net B0	**0.1438** ^**a**^	**0.8654** ^**g**^	**0.7451** ^**i**^	**0.7333** ^**i**^	**64.41** ^**f**^

Again, we used the Wilcoxon signed-rank test to provide greater clarification. Table 3 provides illustrations of the Wilcoxon signed-rank test. By utilizing + and -, the superiority, inferiority, and parity of alternative classifiers concerning EfficientNetB0 are demonstrated. The EfficiientNetB0 classification method statistically outperformed the other 16 classification methods. The EfficientNetB0 model provided the highest performance, according to the results from the remaining models, with a mean accuracy of 86.66%.

**Table 3:** Shows Sign output of different CNN models in wilcoxon sign test concerning EfficientNetB0

Classification Model	Alexnet	Darknet19	DarkNet53	DenseNet201	Googlenet	InceptionResnetV2	InceptionV3	MobilenetV2	Nasnetlarge	NasnetMobile	Resnet18	Resnet50	ResNet101	Vgg16	Vgg19	Xception
Accuracy	-	-	-	-	-	-	-	-	-	-	-	-	-	-	-	-
Sensitivity	-	-	+	+	-	-	-	+	-	-	-	-	-	-	-	-
Specificity	+	+	+	-	+	+	+	+	+	-	-	-	-	-	-	+
Precision	-	+	-	+	-	+	-	-	+	-	-	-	-	-	-	-
FPR	-	+	+	+	-	+	-	-	-	-	-	-	+	-	-	+
F1_Score	-	-	-	-	-	-	-	-	-	-	-	-	-	-	-	-
MCC	-	-	-	-	-	-	-	-	-	-	-	-	-	-	-	-
Kappa	-	-	-	-	-	-	-	-	-	-	-	-	-	-	-	-
Computational Time	-	-	-	-	-	-	-	-	-	-	-	-	-	-	-	-

The performance of EfficientNetB0 was further enhanced by modifying the feature layers of the CNN model.

**Fig. 4:**
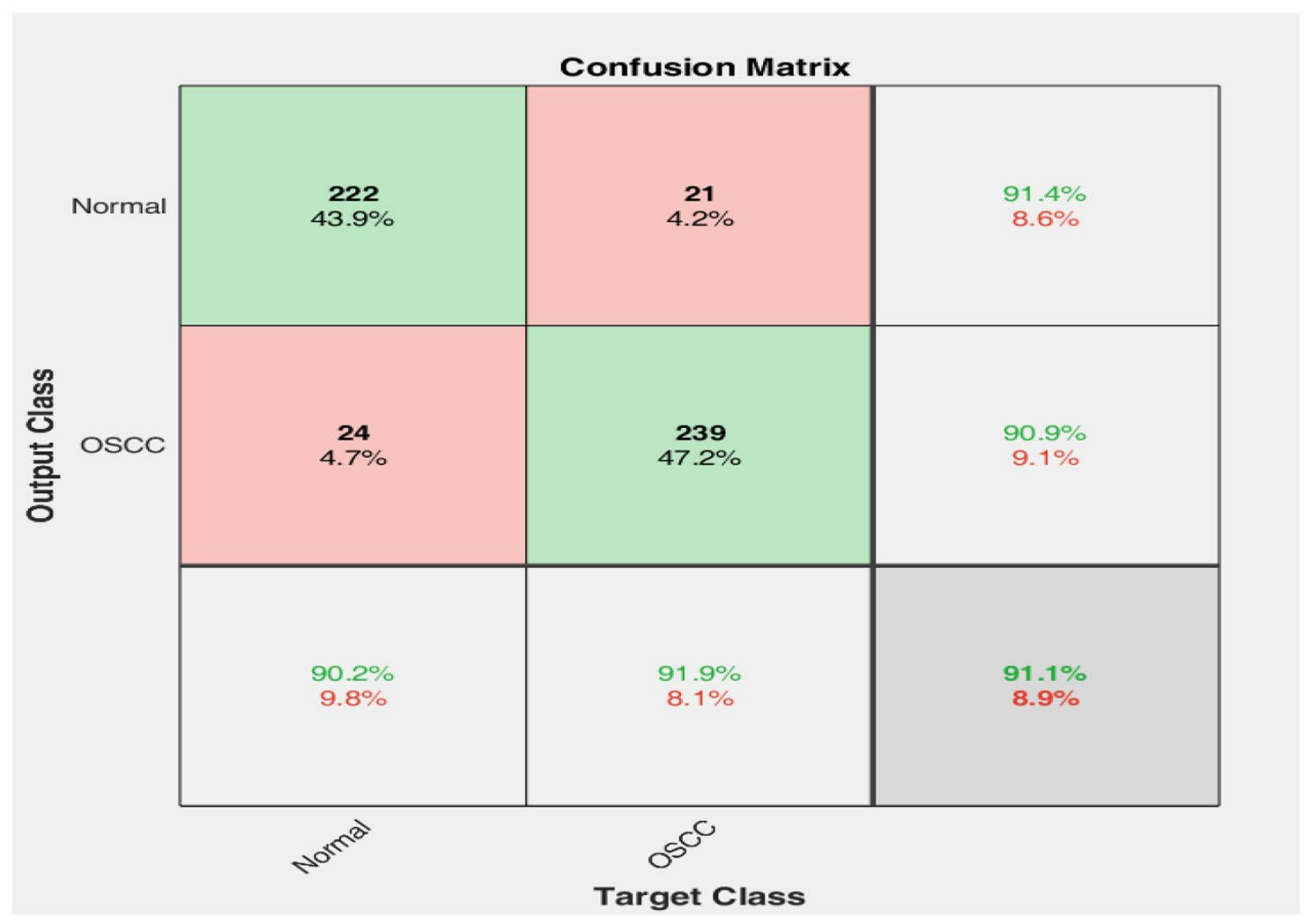
Confusion matrix of improved EfficientNetB0.

The improved EfficientNetB0 was evaluated in terms of the same seven confusion matrix measures. The confusion matrix of the improved EfficientNetB0 is illustrated in Fig. 4.

Hence, the proposed method achieved an accuracy of 91.1%, a sensitivity of 92.2%, a specificity of 91.0%, a precision of 91.3%, an FPR of 1.12%, an F1 score of 92.3%, an MCC of 90.1%, a kappa of 88.8%, and a computational time of 66.41%.

Furthermore, a state-of-the-art comparative analysis was carried out, as illustrated in Table 4.

**Table 4:** Comparison with previous work

Classification models	Accuracy (%)
Gupta et al. [17]	89.30
Song et al. [18]	86.90
G. Forslid et al. [38]	82.39
Rutwik et al. [39]	89.52
Welikala et al. [40]	88.20
Rahman et al. [33]	89.70
H. Wieslander et al. [41]	78–82
Kim et al. [42]	78.10
M. Aubreville et al. [43]	88.30
Shaban et al. [44]	82.39
Proposed model (Improved EfficientNetB0)	91.1

## Conclusion

Recent advances in DL techniques have made it possible to diagnose oral squamous cell cancer (OSCC) automatically, with performance on par with or exceeding that of highly qualified human specialists. In this study, improved DL-CNN models were used to automatically categorize normal and malignant oral histopathology images. A CNN model based on EfficientNetB0 was proposed in this work. For effective OSCC detection, a suggested DL-CNN model was built with the appropriate additional layers, and the candidate models were adjusted using this architecture. Among the other modified models tested, the EfficientNerB0 DL-CNN model achieved an accuracy of 86.66%. Additionally, it was discovered that the results of the suggested work were noticeably better than those of some renowned studies. An accuracy of 91.1%, a sensitivity of 92.2%, a specificity of 91.0%, a precision of 91.3%, an FPR of 1.12%, an F1 score of 92.3%, an MCC of 90.1%, a kappa of 88.8%, and a computational time of 66.41% were attained in the categorization of OSCC histopathological images. Moreover, the proposed model outperformed other CNN models and models used in previous studies. In the future, enhancing the interpretability of the DL-CNN model’s predictions using attention mechanisms and saliency maps will be pivotal, fostering trust among clinicians and facilitating its seamless integration into clinical practice. Additionally, conducting large-scale clinical validation studies and obtaining regulatory approval are paramount steps toward the adoption of this model in real-world healthcare settings. Integration with telemedicine platforms holds promise for extending access to timely OSCC diagnoses, particularly in underserved regions. Moreover, establishing a feedback loop mechanism for continuous model improvement based on real-world performance data will ensure that the DL-CNN model remains adaptive and responsive to evolving clinical needs.

## Data Availability

The datasets generated during and/or analyzed during the current study are available in the KAGGLE repository, https://www.kaggle.com/datasets/ashenafifasilkebede/dataset.
